# Alumni Association of the Chicago Medical College

**Published:** 1871-04

**Authors:** 


### Proceedings of Societies.

### ALUMNI ASSOCIATION OF THE CHICAGO MEDICAL COLLEGE.

The Fourth Anniversary Meeting of this Association was held at 10 A.M., March 14, in the College Hall.

The usual number were present, and the exercises of a varied and interesting nature were conducted with despatch.

Dr. Lyman Ware, of Chicago, class of 1866, obtained the prize of $100 for his essay on "Antagonism between Opium and Belladonna."

The Association again offers a prize of $100, to be governed by the same rules as heretofore. Of the above sum Prof. N. S. Davis donates $50.

The Committee on Prize Essays, Drs. N. S. Davis, E. Andrews, H. A. Johnson, and Walter Hay, were requested to serve another year.

It was moved by Dr. Merriman, and carried, that the Prize Essay and the President's Address be published in THE CHICAGO MEDICAL EXAMINER, that 150 copies be printed in pamphlet form, and a copy sent to each member of the Association.

The officers for the ensuing year are:

*President*, DR. NORMAN BRIDGE, class '68, of Chicago.

*1st Vice-President*, DR. WM. DOUGAL, class '68, Lamont, Ill.

*2d Vice-President*, Dr. GEO. E. LORD, class '71.

*Secretary and Treasurer*, DR. S. A. MCWILLIAMS, class '66, Chicago.

